# Perspectives of Technical Assistance Providers on Nature-Based Solution Practices in North Carolina, USA

**DOI:** 10.1007/s00267-026-02570-6

**Published:** 2026-08-17

**Authors:** Courtney B. Deviney, Rajan Parajuli, Frederick W. Cubbage, Erin O. Sills, Robert E. Bardon, Stephanie Chizmar, Kathleen McGinley

**Affiliations:** 1 Department of Forestry and Environmental Resources, North Carolina State University, Raleigh, NC USA; 2 USDA Forest Service, Southern Research Station, Research Triangle Park, Durham, NC USA; 3 International Institute of Tropical Forestry, USDA Forest Service, San Juan, Puerto Rico, USA

**Keywords:** Ecosystem services, Farm and forest management, Incentive programs, Professional assistance, Landowner outreach

## Abstract

Nature-based solutions (NBS) are increasingly promoted in agricultural and forestry policy, yet their effectiveness depends on technical assistance providers (TAPs) who translate policy guidelines into land management decisions. Drawing on Diffusion of Innovations and Value–Belief–Norm frameworks, this study examines how TAPs’ professional backgrounds, organizational affiliations, regional contexts, and management experiences shape their knowledge, perceived importance, and promotion of NBS. A web-based survey of 101 TAPs in North Carolina was analyzed using Spearman correlations and logistic regression. While 79% of respondents reported familiarity with NBS, only about half of them actively promoted these practices. Adoption pathways varied across sectors. In agriculture, recent management experience was linked to greater emphasis on ecosystem services and higher likelihood of promoting NBS. In forestry, engagement was stronger for establishment and periodic management practices than for timber-oriented activities. These findings highlight the need for sector-specific outreach strategies that account for TAPs’ professional contexts and institutional settings to improve NBS adoption on working lands.

## Highlights

- TAPs act as effective mediators between NBS policy and local implementation.
- A significant “promotion gap” exists: 79% of TAPs are informed, but only 50% actively promote NBS.
- Agricultural TAPs with recent field experience show higher self-reported preparedness to support ecosystem services.
- Timber-harvesting focused forestry TAPs exhibit lower engagement with NBS than those focused on forest establishment.

## Introduction

In recent years, the U.S. forestry and agriculture sectors have increasingly adopted sustainable land management practices such as crop rotation, riparian buffer establishment, erosion control, and post-harvest replanting, to enhance ecosystem health, protect water quality, and sustain ecosystem productivity (Newell et al. 2014, Croft et al. 2021, Nowack 2022). These practices, supported mostly by government agencies, reflect the growing recognition of nature-based solutions (NBS) as effective approaches for addressing environmental degradation while sustaining the flow of ecosystem goods and services (DOI 2022, White House Briefing Room 2022, Thompson et al. 2023). NBS encompass a broad set of practices and strategies, including flood mitigation, carbon management, wetland restoration, soil conservation, and multi-use land management that deliver ecological, economic, and social co-benefits (IUCN 2020). In the U.S., nonprofit organizations have also played a critical role in promoting NBS practices across both agricultural and forested landscapes for broader sustainable development goals and environmental sustainability (NFF 2016, Valutis and Mullen 2000, RTI 2023, AFF 2025).

While NBS are increasingly integrated into conservation policy and funding programs, the broader adoption and implementation of NBS practices face significant challenges due to ambiguity in terminology, varying policy interpretations, a lack of standardized metrics for success, global uncertainty in environmental success metrics, and stakeholders’ limited understanding of NBS (Nature 2017, Davidson et al. 2019, Ruxton et al. 2019, Seddon et al. 2020, Anderson et al. 2021). Similarly, Melanidis and Hagerman (2022) presented two conflicting narratives surrounding NBS in international environmental governance, arguing that the strategic use of definitional ambiguity by proponents and opponents of NBS could produce power asymmetries that ultimately undermine its effectiveness.

Technical Assistance Providers (TAPs), including Extension agents, consulting foresters, natural resource specialists, and loggers act as crucial and trusted intermediaries linking policy frameworks with on-the-ground management (NRCS 2022, CCLR 2024). Given that many NBS-aligned activities are embedded in cost-share and other financial incentive programs, the role of TAPs as an intermediary becomes central to their effective adoption and broader implementation. They provide professional guidance to landowners, help interpret complex program requirements, and assist in the technical design and execution of conservation practices. TAPs have long been recognized as instrumental in fostering landowner participation and ensuring the success of voluntary conservation and climate-smart management programs (Mast 2003, Beach et al. 2005, Ruseva et al. 2014, Snyder and Kilgore 2018). With their influential role in landowners’ awareness, perceived feasibility, and economic assessment of sustainable practices, TAPs effectively mediate the translation of policy goals into measurable landscape outcomes from sustainable land management (Prokopy et al. 2008, Mase and Prokopy 2014).

Despite the critical role of TAPs in implementing NBS practices in working rural lands, little is known about their knowledge, attitudes, and perceptions toward NBS, specifically in the U.S. rural context. Most empirical studies on climate-related beliefs and behaviors of consultants and educators have focused on topics such as sustainable agriculture, carbon markets, or climate-smart forestry (Knoot et al. 2015, Yang et al. 2024), but only few have explicitly framed their analysis around the NBS concept. This gap partly reflects the recent adoption of the term “NBS” itself, as well as the fact that research on NBS has been mostly concentrated in urban contexts (Van Der Jagt et al. 2023, Dunlap et al. 2024). Understanding TAPs’ knowledge, values, and organizational contexts from the southeastern United States is thus essential for designing effective outreach and policy mechanisms that advance widespread adoption and implementation of NBS practices for achieving long-term resilience and sustainability.

NBS hold promise for the southeastern U.S., where forests act as major carbon sinks through reforestation, afforestation, and improved forest management (Janowiak et al. 2014), and agricultural systems contribute to carbon storage via reduced tillage, cover cropping, and agroforestry (Smith et al. 2019). These practices exemplify how NBS integrate climate mitigation with co-benefits such as biodiversity conservation, soil fertility, and water regulation (Griscom et al. 2017, IUCN 2020). For example, in North Carolina, state-level strategies now aim to reduce carbon emissions and enhance ecosystem resilience by promoting climate-aligned land management across public and private lands (NCDEQ 2024, NCORR 2024, NCDEQ 2025). Despite this policy momentum, achieving widespread NBS adoption will depend on the effectiveness of local intermediaries, who interpret and translate these strategies into tangible actions on farms and forests.

The main contribution of this study is twofold. First, there is a lack of systematic understanding of how TAPs conceptualize and promote NBS within their professional roles. Although TAPs are often key influencers of landowner behavior, research rarely investigates their familiarity with NBS frameworks or how their institutional settings shape engagement. Second, no studies to our knowledge have compared TAPs from the agricultural and forestry sectors in their understanding, perceived importance, and promotion of NBS. This distinction is theoretically and practically significant because these two groups operate under different management objectives, technical priorities, the level of engagement required, and financial setups. Forestry TAPs typically emphasize long-term ecological outcomes such as carbon sequestration, biodiversity enhancement, and forest regeneration, whereas farm TAPs often prioritize short-term outcomes related to soil health, productivity, and risk mitigation (Snyder et al. 2008, Ruseva et al. 2014). These orientations influence not only the kinds of practices they recommend but also how they frame conservation benefits to landowners. For example, forestry specialists may stress ecosystem services and landscape-scale resilience, while agricultural consultants are more likely to focus on financial returns or immediate yield improvements. Moreover, climate adaptation is often integrated more deeply into forestry guidance, while agricultural TAPs tend to respond to immediate, production-related stressors such as drought or pest outbreaks (Janowiak et al. 2014).

Understanding TAPs’ environmental beliefs and organizational contexts is essential for predicting their behaviors and improving the design of technical assistance and education programs (Heberlein 2012). This study aims to assess TAPs’ knowledge, perceived importance, and promotion of NBS-related practices, and examine whether stronger beliefs of TAPs in NBS importance are associated with higher levels of active promotion with their clients. We hypothesize that (a) TAPs’ professional background and organizational sectors significantly influence the perceived importance of NBS practices; (b) these characteristics also shape their knowledge, preparedness, and current actions in promoting NBS; and (c) TAPs who perceive NBS as highly important are more likely to actively advocate for and implement them. Findings of this study contribute to the growing literature on land-use governance and sustainability transitions by highlighting the underexplored role of TAPs in operationalizing NBS. Importantly, distinguishing between forestry and agricultural service providers advances theoretical understanding of how professional identities and institutional environments mediate the adoption of nature-based climate strategies in rural landscapes. Additionally, while this study focuses on a single North Carolina case, the findings offer broadly transferable insights for sustainable land management through NBS across diverse regional and international contexts.

## Methodology

### Survey Instrument

This study utilized a survey instrument informed by two foundational theories: the Diffusion of Innovations (DOI) and the Value–Belief–Norm (VBN) frameworks. The DOI theory informed the development of questions aimed at assessing perceptions of innovation attributes such as compatibility and complexity, as well as the influence of social networks in shaping adoption patterns (Rogers 2003). For example, the survey items explored how TAPs view the alignment of climate-smart practices with their workflows and whether they consult peers when determining what practices to promote. Conversely, the VBN framework facilitated the operationalization of the value-belief-norm causal chain, utilizing Likert-scale items to quantify pro-environmental motivations (Stern et al. 1999). Together, these theoretical frameworks provided actionable insights pertinent to enhancing service provision and engaging landowners in climate-smart land management.

A web-based survey consisting of 28 questions was administered to 960 TAPs working with forest and farmland owners in North Carolina. The objective was to evaluate perceptions and engagement levels regarding carbon management, climate-smart practices, nature-based solutions (NBS), replanting initiatives, and the barriers encountered by TAPs. Data collection occurred from April 12 to August 9, 2024, following the Dillman Tailored Design Method (Dillman 2011) and drawing on DOI and VBN theories. Participants were estimated to require approximately 20 minutes to complete the survey, which targeted individuals aged 18 and older involved in providing technical services for forest or agricultural land management. The survey was organized into three sections: “General Background,” “Nature-Based Solutions, Tree Planting, and Climate-Smart Management,” and “Demographics.”

To ensure all participants understood what we meant by NBS, we provided this definition in the survey instrument before they consented to participate in the survey:

“We are conducting this survey to gauge your opinion as a forest or farmland technical assistance provider on management techniques that fall under the term “nature based solutions”. Nature based solutions (NBS), which have traditionally been termed conservation practices, have been expanded for a broader array of land management applications, such as erosion control, water management, soil health, wildlife and biodiversity, carbon storage management, and forest planting. Rural land management incentives have been developed since at least the 1960s Soil Bank Program to support and reward landowners that participate in farm and forest conservation. Throughout this survey you may see the term “climate smart management” or “climate smart forestry” being used. This term encompasses the use of nature based solutions, that are listed above, being used to help offset or assist with lowering carbon and other greenhouse gas emissions.”

The General Background section comprised nine questions designed to identify respondents’ affiliations within the natural resources sector and the extent of their management activities. To minimize respondents’ burden and enhance response rates, mixed types of questions were utilized, integrating categorical variables (e.g., organizational size) with continuous variables (e.g., acreage managed) (Suski 1988, Fowler 2013, Geisen 2023). Multiple-choice questions facilitated ease of recall and privacy, while open-ended questions provided opportunities for more detailed, nuanced responses, thereby reducing the likelihood of overgeneralization. Furthermore, multiple-response questions concerning fields, sectors, work focus, and certifications increased flexibility in self-categorization and yielded richer detail (Fowler 2013, Geisen 2023). This comprehensive survey design afforded a clearer understanding of TAPs’ sectors of work, organizational scales, and workloads.

The section entitled “Nature-Based Solutions, Tree Planting, and Climate-Smart Management” comprised twelve questions aimed at evaluating TAPs’ beliefs, levels of support, and engagement with NBS, carbon management strategies, and climate adaptation efforts. The questions were intentionally designed using categorical frameworks to capture complex opinions, identify knowledge gaps, and assess belief patterns, while mitigating response biases. The incorporation of both multiple-choice and multiple-response formats facilitated participant engagement and enhanced data collection (Fowler and Cosenza 2009, Fowler 2013, Geisen 2023). Likert-scale items were employed to gauge TAPs’ attitudes (Likert 1932, Suski 1988, Geisen 2023). The Demographics section gathered comprehensive data on gender, ethnicity, race, education level, years of service, experience in natural resources, geographical region, and age. This information was utilized in the analysis of how individual backgrounds may influence professional perspectives and decision-making processes, thereby providing insights into the role of personal values in shaping TAPs’ engagement with NBS.

### Survey Administration

This survey encompassed a diverse group of TAPs, characterized as individuals offering technical, hands-on, or advisory services to landowners. The study included both public and private service providers. Contact information was compiled from various publicly accessible databases, such as the registered foresters list from the NC Board of Foresters, the NC Forest Service’s county-based technical assistance directories, NC FarmLink, and the USDA Farmers Service Provider database (ACF 2024, NC Extension 2024, NCBORF 2024, NC FarmLink 2024, USDA 2024, NCFS 2025). We aimed to reach out to TAPs for both forest and farm landowners, which is why we pulled from both forest and farm TAPs databases, but we allowed the TAPs to report the field of work that they felt more strongly aligned with during the survey. It is possible that individuals who reported working with farmland may occasionally still manage or provide advice for wooded areas. Since we put no parameters around what an individual had to participate in to be a specific TAPs type, it was subjective to how they identify from their own work experience.

A pretest involving seven participants (two farmland managers, an extension agent who works with forest and farm landowners, a private timber company employee, a state forester, a consulting forester, and an extension agent who works in forestry outreach) was conducted to assess the clarity, timing, and flow of the survey instrument. Respondents completed the survey on either a mobile device or a laptop, and their feedback prompted minor revisions in design and formatting. The finalized survey was administered online via Qualtrics, with the entire survey administration, data collection, and security protocols reviewed and deemed exempt by the NC State University Institutional Review Board (IRB #25932). The distribution process began with an introductory email, followed by the survey link sent the subsequent day. Reminder emails were dispatched bi-weekly over a one-month period, culminating in a final reminder that included the survey link.

**Fig. 1:**
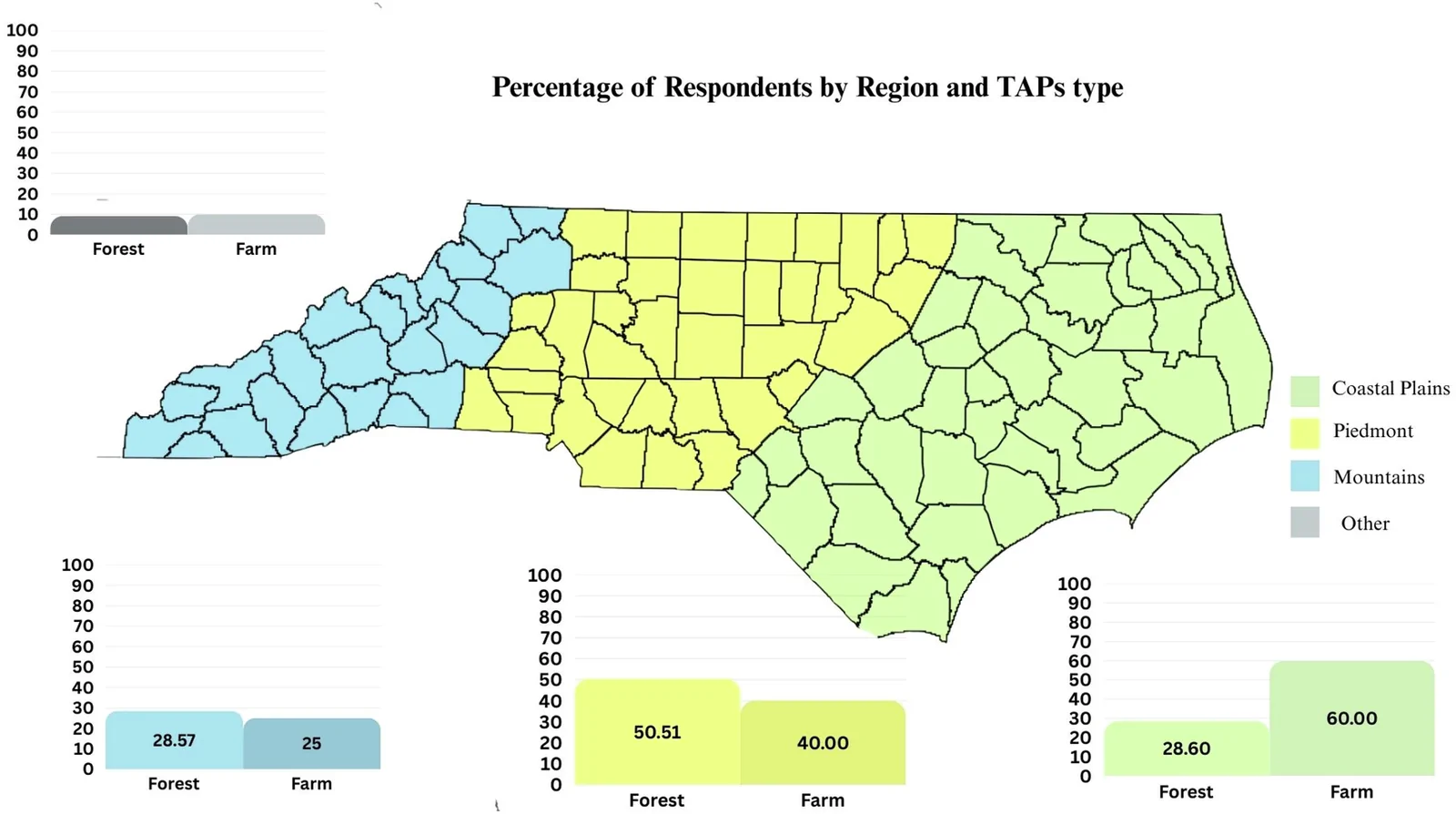
illustrates the response rates of all TAPs across various physiographic regions in North Carolina

We sent 81 surveys to individuals from farm databases and 879 to individuals from the forestry contacts for a total of 960 surveys. The survey achieved a response rate of 21% and a completion rate of 91%, resulting in a total of 180 responses. Furthermore, 126 emails were reported as undeliverable, likely due to outdated or inaccurately reported addresses, hindering further outreach efforts. The final response list comprised 101 retained respondents, representing 10.5% of the estimated total TAP population within the state as 79 surveys were deemed incomplete and excluded from subsequent analyses. We had 20 individuals who identified as farm TAPs and 81 who identified as forestry TAPs. Figure 1 below provides an overview of the response rate by region. The three regions depicted are the three major physiographic regions of North Carolina. The mountain region is comprised of chains of the Appalachian mountain range in the western part of the state. The Piedmont region is the central stretch and includes rolling hills, fertile soils, and dropping elevation towards the east. The coastal plains are the largest eco region, which is the flattest eco region, consisting of both rolling hills and swamps and tide water touching the Atlantic Ocean. While this study is limited to North Carolina, the state shares similar climate, socio-economic, cultural, land ownership, and policy characteristics with much of the broader southern U.S. region, suggesting the findings may have broader regional relevance.

### Statistical Analysis

The analysis began with a detailed examination of the dataset to characterize the respondents and discern preliminary patterns. These statistical measures illustrated the distribution of demographic characteristics, work experiences, and views on climate and carbon management practices among TAPs and their perceived landowner clients. This descriptive foundation informed subsequent analyses and provided context for interpreting significant relationships. Table 1 lists the explanatory variables used in the analysis and their key summary statistics.

**Table 1:** Explanatory variables and their descriptions along with key summary statistics

	Definition	Mean (Std. Deviation)
*Education*	The level of education a TAP completed (1= less than 12^th^ grade, high school or GED, some college, associate’s degree, bachelor’s degree, 6= advanced degree)	3.98 (0.58)
*Age*	Age of TAPs converted from categories to midpoints (24 years, 38 years, 53 years, 68 years, and 80 years)	51.63 (15.37)
*Years employed*	Number of years TAPs has worked as a TAPs (0.5 years, 3.5 years, 7.5 years, 12.5 years, 17.5 years)	13.24 (5.73)
*Regional Information*	The regional location the TAPs work in Piedmont, Coastal Plain, and Mountains. (Binary variables 1= yes, 0 = No)	0.462 (0.49) 0.473 (0.50) 0.275 (0.44)
*Field of work*	The field of agriculture or forestry the TAPs work in.	
*Focus of work*	The primary focus of work a TAPs is employed in	
*Sector of work*	The sector a TAPs is employed in	
*Organization size*	The size of the organization based on number of employed individuals. (1, 6.5, 15, 25, 35, 45, 60 + )	19.25 (15.37)
*Crop and pastureland managed*	Average amount, in acres, of crop and pastureland managed by TAPs	3763.28 (17960.08)
*Forest land managed*	Average amount, in acres, of forest land managed by TAPs	42461.31 (153695.5)
*Expanded carbon markets*	Support of using expanded carbon markets as the most effective approach to NBS for reforestation or afforestation by landowners they currently work with (1=yes, 0=otherwise)	0.41 (0.494)
*Replanted in past five years*	Average amount of land, in acres, advised by TAPs that has been replanted annually in the past five years	7556.41 (42479.6)
*Afforested in past five years*	Average amount of land, in acres, advised by TAPs that has been afforested annually in the past five years	103.04 (310.30)
*Regenerated naturally in past five years*	Average amount of land, in acres, advised by TAPs that has been naturally regenerated annually in the past five years	5009.41 (41262.1)
*Harvested in past five years*	Average amount of land, in acres, advised by TAPs that has been harvested annually in the past five years	2545.2 (9092.20)

This study investigates the relationships among TAP backgrounds, their organizational affiliation or sector of work, and their perceptions on NBS and climate-smart practices. Specifically, we aim to elucidate how these contextual factors shape the significance that TAPs ascribe to climate management and NBS as well as their levels of support and advocacy for such practices. A detailed description of these outcome variables is provided in Table 2. Furthermore, we examined the correlation between perceived importance ratings and both promotional behaviors and landowner interest perceptions, utilizing Spearman correlation analysis. We established a robust approach to assess the intricate dynamics among individual values, institutional contexts, and TAP engagement with NBS practices by employing a dual-theoretical framework in our survey.

**Table 2:** Outcome variables and their description along with key summary statistics^a^

*Variable*	Description	Mean (Std. Deviation)
*NBSknowledge*	If TAPs report having knowledge enough to promote NBS as a management option to their landowners (Binary: 1= Yes, 0= Otherwise)	0.79 (0.44)
*NBSpromoting*	If the TAPs reported they have knowledge enough and are currently promoting NBS as a management option to their landowners in (Binary: 1= Yes, 0= No)	0.51 (0.50)
*LOIPFM*	The level of importance TAPs ascribed to *Periodic Forest Management (e.g., thinning, invasive species control, prescribed fire)* to TAPs on a categorical scale, 1–4	3.81 (0.51)
*LOIPFMWCP*	The level of importance TAPs ascribed to *Farm Management with Conservation Practices* (*e.g., cover crop, no till cropping, hardpan breakup, tiling, and agroforestry)* on a categorical scale, 1–4	3.45 (0.69)
*LOIPES*	The level of importance TAPs ascribed to *Farm Ecosystem Services (e.g., wildlife cover, stream buffers and restoration, wetlands restoration, runoff, and impoundments*) on a categorical scale, 1–4	3.3 (0.66)
*LOISI*	The level of importance TAPs ascribed to *Forest Site Improvement* (*e.g., wildlife habitat, water management, erosion control, forest wetland management*) on a categorical scale, 1–4	3.33 (0.69)
*LOIFGM*	The level of importance TAPs ascribed to *Forest General Maintenance e.g., road maintenance, fire line maintenance, trail maintenance* on a categorical scale, 1–4	3.32 (0.69)
*LOIFE*	The level of importance TAPs ascribed to Forest *Establishment* (*e.g., tree planting on forest or agriculture land*) on a categorical scale, 1–4	3.26 (0.83)
*LOIFMC*	The level of importance TAPs ascribed to Farm *Management for Carbon* (*e.g., interplanting, low carbon product selection*) on a categorical scale, 1–4	2.37 (0.83)
*LOIMCO*	The level of importance TAPs ascribed to *Management for Forest Carbon Offsets* (*e.g., longer rotation, interplanting*) on a categorical scale, 1–4	2.22 (0.95)

These variables were created by dividing a question on TAPs knowledge and if they felt equipped to promote NBS and if that knowledge led to them currently promoting NBS practices.

All Variables but NBSknowledge and NBSpromoting are on a 4 point scale (1 = not important, minimally important, somewhat important, 4 = very important).

To prepare categorical variables such as age, organization size, and years of employment for quantitative analysis, we assigned midpoint values to each category. These numeric estimates enabled us to analyze these variables as continuous predictors in regression models, facilitating the interpretation of their impacts on perceived importance levels regarding various forest management practices. Additionally, due to our small sample size we could not afford to use separate categorical variables for each level. We also opted to divide our sample into forest and farm TAPs. We divided the sample due to the differing nature of the two groups; for example, forest TAPs work on a much longer timeline than those who work in farming, they also vary vastly in the goals they aim to achieve. Dividing the two groups allowed us to identify perspectives without the unknown impact of these two groups’ existing differences.

Due to the limited sample size of farm TAPs, we estimated Spearman correlations to examine statistical relationships. The Spearman correlation test identifies monotonic associations between ordinal or non-normally distributed continuous variables, with the sign and magnitude of the coefficient indicating both the direction and strength of the association (Laerd Statistics 2018). Given the data’s distributional characteristics and the exploratory nature of the analysis, we estimated a logistic regression model, as the dependent variable exploring whether the respondent feels well-informed on NBS options was binary. This mixed-use technique estimates the influence of predictors while respecting the limitations of the variation in response size. The regression model is presented below:

$${Pi}=log \left(\,\frac{1}{1-P}\,\right)=\beta 0+\beta 1\,X1i+\beta 2\,X2i+\beta 3\,X3i+\beta 4\,X4i$$

*Pi:* The probability a TAP has knowledge of NBS

β0: The intercept term

X1i: Background variables

X2i: Regional variables

X3i: Organization affiliation (i.e., the organization or entity the respondent worked for) and size variable

X4i: Opinion variables

## Results

Among the 101 respondents working as farm and forestry TAPs in NC, the majority identified as male (85.23%), with 9.09% identifying as female and 5.68% opting not to disclose their gender. The age distribution indicated that most TAPs (34.48%) were aged between 46 and 60 years, followed by 28.74% who were 61–75 years old and 21.84% aged 31–45. A minimal proportion (3.45%) were aged 76 or older. A more detailed distribution of respondents’ areas of focus, field, and sector can be found in Table *3* below.

**Table 3:** Division of TAPs types by their field, focus, and sector of work

Sector of work		Field of work		Focus of work	
Private farmland or forest land profession	53.80%	General forest management	73.10%	Timber management	70.20%
Forest or farm consultant	33.30%	Timber Harvesting	45.20%	Logging/harvesting	41.50%
State Government	19.40%	Forest restoration management	35.50%	Site preparation and tree planting	36.20%
Federal Government	7.50%	Forest Health	25.80%	Forest restoration	33.00%
Private sector Timber Investment Management Organization (TIMO)	7.50%	Soil and Water conservation or soil health	10.80%	Fire management	17.00%
Non-government organization	5.40%	Wildlife management	10.80%	Wildlife	12.77%
Nonprofit organization	5.40%	Landowner outreach	9.70%	Forest pest and disease management	10.60%
Private sector forest or farm Real Estate Investment Trust (REIT)	3.20%	Farmland Management	8.60%	Soil health and erosion control	9.60%
Other Government	1.10%	Forest conservation	7.50%	Water quality and quantity control	9.60%
		Wetland mitigation	3.20%	Row crops	5.30%
				Fruit or Christmas trees	2.10%
				Pasture management	2.10%

*n* = 101. The percentages may not add up to 100% as TAPs could select up to three variables for field, sector, and focus of work.

### TAPs opinions on farm NBS-based practices

**Fig. 2:**
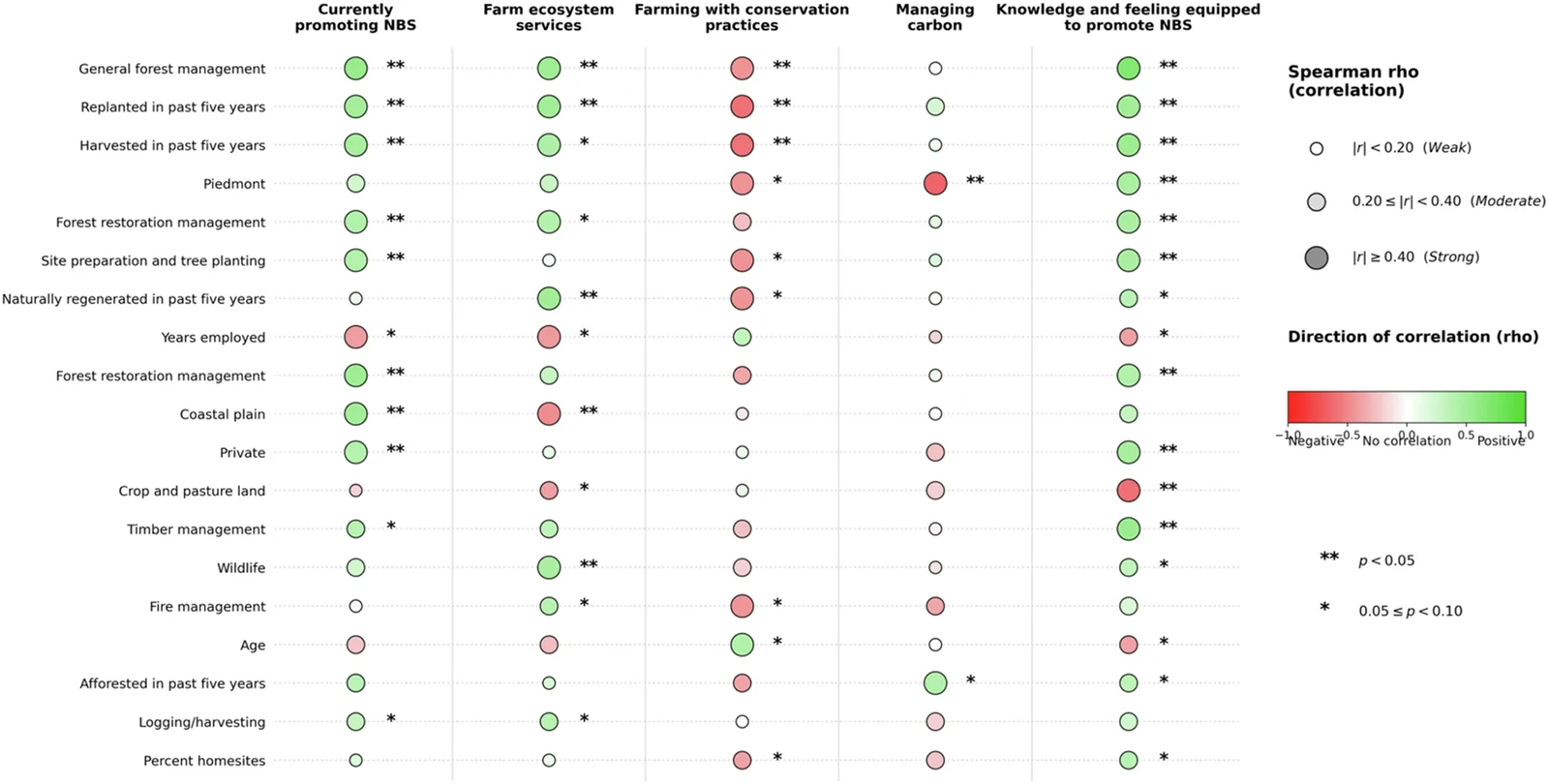
Spearman correlation results for Farm TAPs detailing the relationship between strength and direction for the TAPs level of importance of practices and their confidence promoting NBS practices in relation to the TAPs background, focus of work, and organization structure. The color denotes relationship direction and size of the circle denotes strength; two asterisks represent a statistical significance at 5% and one asterisk represents the 10% level of significance

Given the diverse backgrounds of TAPs, this study assessed the extent to which their professional experiences and the organizational sectors of their workplace influence their perceptions on the relevance of NBS practices. Additionally, we examined how these factors impact their confidence in endorsing NBS practices to landowners. Our analysis of the correlations between TAPs’ professional attributes and their perceived significance of various farm-focused NBS management practices identified several statistically significant relationships (see Fig. 2)^1^.

In the context of farm conservation practices, significant negative associations were identified with the Piedmont region (*p* = 0.052). Additionally, TAPs focused on General Forest Management (*p* = 0.047), Site Preparation and Tree Planting (*p* = 0.052) or Fire Management (*p* = 0.066) exhibited negative relationships with the level of importance assigned to these practices. We also found a negative association between the proportion of homesites or buildings on managed farmland and its perceived importance (*p* = 0.097). Stronger negative correlations were observed with the number of acres replanted by clients over the past five years (*p* = 0.007), the number of acres naturally regenerated by clients (*p* = 0.054), and the number of acres harvested for clients annually (*p* = 0.008). These findings suggest that TAPs associated with certain geographic regions or land uses tended to place lower importance on conservation practices. In contrast, a positive relationship was detected between the level of importance placed on farm conservation and increasing TAP age (*p* = 0.066).

For farm ecosystem services, significant negative associations were observed for TAPs located in the Coastal Plains region (*p* = 0.038), those specializing in Row Crops (*p* = 0.038), and those focusing on Soil Health and Erosion Control (*p* = 0.081). Negative correlations were also detected between the perceived importance of ecosystem services and both the total acreage of cropland and pastureland managed (*p* = 0.085) and the percentage of cropland managed (*p* = 0.043). Conversely, positive associations were evident for TAPs concentrating on General Forest Management (*p* = 0.018), Forest Restoration (*p* = 0.071) and for those working with Fruit or Christmas Trees (*p* = 0.092).

At the land-use level, TAPs managing larger shares of pastureland reported assigning higher importance to ecosystem services (*p* = 0.060). Stronger positive associations were observed for the scale of activities carried out by clients, including annual increases in replanted tree acreage (*p* = 0.024), naturally regenerated acres (*p* = 0.024), and acres harvested (*p* = 0.053). Taken together, these results indicate that TAPs’ valuation of ecosystem services varies systematically by geographic context, production system, and client land-use activity.

Investigating TAPs’ knowledge and perceived preparedness to promote NBS practices, several significant and marginal associations were identified. Positive associations were observed for TAPs engaged in General Forest Management (*p* = 0.0002), Forest Establishment (*p* = 0.072), Site Preparation and Tree Planting (*p* = 0.072), and Forest Restoration (*p* = 0.072). Clients’ replanting activities in the past five years (*p* = 0.013) and acres harvested (*p* = 0.008) also showed significant positive correlations with TAP preparedness. Conversely, TAPs whose work emphasized Row Crops demonstrated a strong negative relationship with preparedness (*p* = 0.016), as did those who placed higher importance on Water Management (*p* = 0.069). Most notably, TAPs who prioritized Recreation reported significantly lower levels of preparedness (*p* = 0.003).

When examining actual engagement in NBS promotion, significant positive relationships were identified for TAPs managing larger areas of replanted land (*p* = 0.023) and land harvested in recent years (*p* = 0.030) as well as TAPs working in General Forest Management (*p* = 0.003). Positive though weaker associations were also observed for Forest Establishment (*p* = 0.117) and Site Preparation/Tree Planting (*p* = 0.029). By contrast, years of employment were negatively associated with NBS promotion (*p* = 0.069), indicating that longer-tenured TAPs reported lower promotion levels compared to newer staff.

### TAPs opinions on forest NBS based practices

**Fig. 3:**
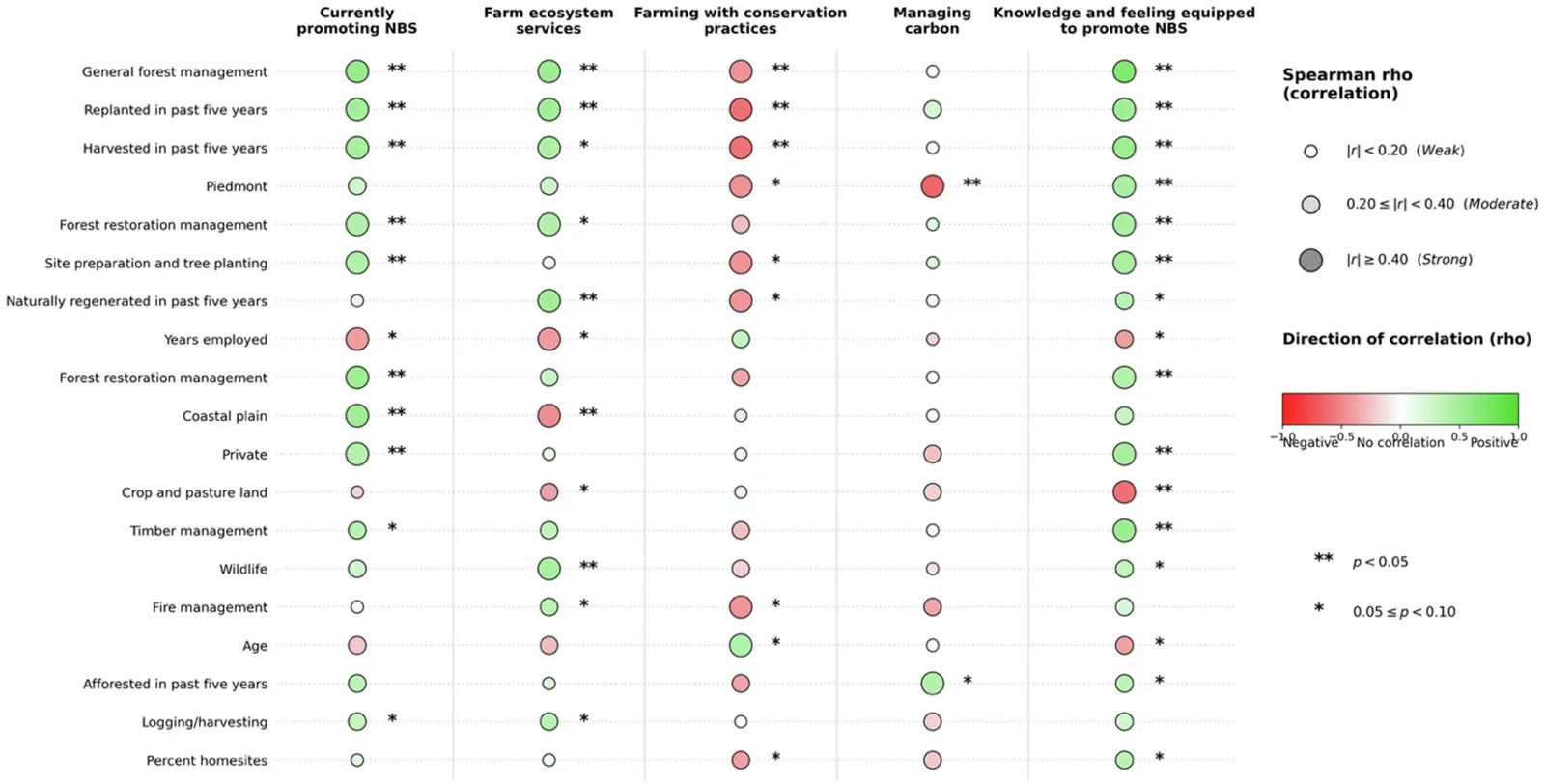
Spearman correlation results for forest TAPs detailing the relationship between strength and direction for the TAPs level of importance of practices and their confidence promoting NBS practices based on the type of TAPs and location of work. The color denotes relationship direction and strength; two asterisks represent a statistical significance at 5% and one asterisk represents the 10% level of significance

We also examined the viewpoints of TAPs on forest-oriented NBS practices, identifying several key associations and factors describing TAPs’ opinion and current knowledge of NBS. The results of the Spearman correlation analysis, presented in Fig. 3,^2^, highlight both the strength and direction of the relationships between TAP characteristics and their level of importance on NBS, as well as the correlation between TAP attributes and their confidence in executing NBS practices.

TAPs’ involvement in specific management domains and their perceived importance on forest establishment revealed several significant relationships. TAPs focusing on soil health and erosion control (*p* = 0.004) demonstrated a strong positive correlation, as did those involved in water quality initiatives (*p* = 0.086), giving higher levels of importance for forest establishment practices. Conversely, significant negative correlations were observed for TAPs engaged in logging/harvesting (*p* = 0.080) and those operating as private farm or forest professionals (*p* = 0.030). Regarding the reported importance of periodic forest management^3^ across various TAPs descriptors, only one significant relationship was identified: TAPs operating in the coastal plain region (*p* = 0.038).

TAPs’ involvement in specific management domains with respect to general forest maintenance yielded seven significant results. TAPs situated in the coastal plain rated general forest maintenance at higher levels of importance (*p* = 0.006), alongside those identified in the field of forest conservation (*p* = 0.092) or other areas (*p* = 0.029). Additionally, professionals identifying as working for a TIMO assigned greater importance to general forest maintenance (*p* = 0.009). Conversely, individuals involved in landowner outreach regarded general forest maintenance as less important (*p* = 0.009), with similar negative associations noted for non-governmental organizations (*p* = 0.100) and wildlife-focused roles (*p* = 0.062).

Three significant relationships were identified concerning site improvement activities. TAPs from the Piedmont region rated the importance of site improvement considerably lower (*p* = 0.013), alongside private farm or forest professionals (*p* = 0.037). In contrast, professionals engaged in forest restoration assigned a higher importance to site improvements (*p* = 0.084). Regarding the significance of carbon offset management, we identified four key relationships. TAPs from the Piedmont region rated this aspect as significantly less important (*p* = 0.025), as did those involved in logging or harvesting activities (*p* = 0.028). Conversely, individuals focusing on forest health (*p* = 0.049) and particularly those engaged in forest restoration (*p* = 0.000) rated carbon offset management as significantly more important.

In evaluating the factors associated with TAPs’ involvement in specific management domains and TAPs’ knowledge and confidence in promoting NBS, several significant and marginally significant trends emerged. TAPs who placed higher importance on forest establishment were significantly more likely to feel equipped to promote NBS practices (*p* = 0.044), aligning with earlier findings that highlight the relevance of reforestation in shaping practitioner confidence. Marginally significant positive associations were also noted for those emphasizing site improvement (*p* = 0.090) and general forest maintenance (*p* = 0.073), suggesting that traditional silvicultural domains may foster greater familiarity with NBS-related concepts. While few factors were significantly associated with current NBS promotion, an emphasis on soil health and erosion management (*p* = 0.073) positively impacted the level of importance.

To further investigate TAPs’ opinion on NBS practices, we employed logistic regression to assess the factors describing TAPs’ current knowledge of NBS practices so that they feel well-informed and equipped to promote these practices with their clients (Table 4). Several TAPs characteristics and regional variables are found to be statistically significant. TAPs respondents from the Piedmont region are significantly and positively associated with their knowledge of NBS practices (*p* = 0.039), suggesting that compared to the TAPs from other regions, professionals working in the Piedmont region are more likely to feel well-informed and knowledgeable about NBS practices. Forest acreage was also significantly and positively associated with feeling equipped to promote NBS (*p* = 0.022), indicating that those TAPs who work with larger acreage of forestland are more likely to be well-informed on NBS practices. Similarly, TAPs who work as a private consultant with farm and forest landowners are also found more likely to be well-informed on NBS practices (*p* = 0.045).

**Table 4:** Logistic regression results of the factors describing the Technical Assistatnce Providers’ current knowledge and information on nature-based solution practices *n* = 81

Variable	Coefficient	Robust standard errors	*P* value
Primary Focus of Work Logging/Harvesting (1 = yes)	–3.300	1.162	0.005
Field of Work General Forest Management (1 = yes)	–3.334	1.314	0.011
Forest Acres (natural logarithm)	0.452	0.197	0.022
Piedmont (1 = yes)	2.464	1.192	0.039
Sector of Work: Consultant (1 = yes)	1.667	0.830	0.045
Expanded Carbon Markets (1 = yes)	–1.899	0.954	0.046
Age	0.423	0.401	0.291
Education	0.402	0.463	0.386

In contrast, respondents whose focus of work was general forest management (*p* = 0.011) or whose primary focus was logging or harvesting (0.005) were less likely to feel knowledgeable on forestry NBS practices. Further, TAPs’ support for using expanded carbon markets was significantly negatively associated with their perceived knowledge and feeling of well-equipped to promote NBS (*p* = 0.046). Education and age were found not to be significantly associated with TAPs’ perceived knowledge of NBS practices.

As demonstrated in Table *5*, we found four significant relationships between level of importance for forestry based NBS practices and whether TAPs are currently promoting NBS practices in general. TAPs who assigned higher importance ratings to forest establishment (*p* = 0.068), periodic forest management (*p* = 0.014), site improvement activities (*p* = 0.010), and carbon offset management (*p* = 0.046) were significantly correlated with their current promotion of NBS practices.

**Table 5:** Spearman correlation results for TAPs detailing the relationship (strength and direction) for the TAPs’ reported level of importance of practices, and if they are currently promoting NBS practices to the landowners they serve

Perceived importance	*n*	*Spearman’s rho (p value)*
Level of importance on site improvement	79	0.292 (0.010)
Level of importance on periodic forest management	80	0.276 (0.014)
Level of importance on forestry carbon offsets	78	0.227 (0.046)
Level of importance on forest establishment	77	0.2094 (0.068)
Level of importance on general forest maintenance	79	−0.032 (0.777)
Level of importance on farm conservation practices	20	−0.191 (0.418)
Level of importance on farm ecosystem services	20	0.126 (0.593)
Level of importance on farm carbon offset	19	0.066 (0.782)

## Discussion

This study examined how forestry and farm TAPs perceive and promote various NBS practices in North Carolina, focusing on their confidence, perceived importance of specific practices, organizational sector, and the geographic and programmatic scope of their work. As public and private agencies increasingly look to NBS to address climate mitigation, water quality, biodiversity conservation, and community resilience, TAPs play a central role in translating science and policy initiatives into real-world implementation. Our findings reveal substantive differences in how TAPs engage with NBS, driven by their professional background, the structure of their organization, and the types of assistance they routinely provide. These results highlight important insights and the current state of assistance providers for strengthening Extension, outreach, and policy strategies aimed at expanding NBS adoption across diverse landownerships in North Carolina and beyond.

### Influence of TAPs’ Professional Background and Organizational Sectors on the Perceived Importance of NBS Practices

Across both Farm and Forestry TAPs, regional contexts are consistently correlated with NBS priorities. Piedmont TAPs emphasized conservation- and carbon-oriented strategies, while Coastal Plain TAPs placed greater importance on ecosystem service and general maintenance practices, reflecting regional differences in urbanization, flooding risk, soil erosion, and disturbance regimes (Butler and Leatherberry 2004, Dia et al. 2011, Anderson et al. 2021, Hovis et al. 2021, White et al. 2022).

Organizational characteristics and professional specialization are also related to priorities across both groups. Government-affiliated TAPs and those operating within larger organizations generally placed higher importance on establishment and management practices, reflecting institutional mandates and resource capacity. Additionally, recent land-use history moderated perceived importance across contexts: practices were deprioritized when recently implemented and emphasized when land-use change or afforestation was ongoing. Together, these shared patterns underscore the importance of tailoring NBS outreach and incentive design to regional conditions, organizational size, and land-use trajectories.

### Farm TAPs

Farm TAPs’ priorities for farm-based NBS differed primarily by type of management focus and land-use activity. TAPs working in forest or wildlife management assigned lower importance to farm conservation but higher importance to ecosystem service practices, reflecting alignment with habitat- and ecosystem-oriented objectives. Negative associations between ecosystem service importance and both crop/pasture management and organizational size suggest that production-oriented operations may deprioritize ecosystem services. In contrast, TAPs engaged in more dynamic land management activities reported higher ecosystem service importance. Experience-related differences were also evident. Older TAPs placed greater value on conservation, consistent with evidence linking professional experience to stronger conservation preferences (Fazey et al. 2006). TAPs with substantial replanting experience placed lower importance on conservation but greater importance on ecosystem services, indicating that reforestation objectives shift the relative salience of farm-based NBS strategies.

### Forestry TAPs

For Forestry TAPs, professional role and recent land-use activities were the dominant drivers of NBS priorities. TAPs engaged in farmland management, soil health initiatives, or government service placed higher importance on forest establishment, reflecting a focus on regeneration and long-term stand development (Knoot and Rickenbach 2011). Outreach-focused TAPs placed lower importance on periodic and general forest maintenance practices. Recent management history further shaped priorities. TAPs serving landowners who had recently replanted were less inclined to emphasize forest establishment, suggesting that establishment needs had already been met, while TAPs engaged in afforestation efforts placed higher importance on establishment. Organizational scale also mattered: larger organizations were more likely to prioritize forest establishment, likely due to broader mandates and greater resource capacity. Demographic variables such as age, experience, and education showed mixed significance, consistent with prior findings (Joshi and Arano 2009). Government employment was generally associated with higher perceived importance of forest management activities, particularly forest establishment and periodic maintenance.

### Influence of TAPs’ Professional and Organizational Contexts on NBS Knowledge, Preparedness, and Current Actions in Promoting NBS

Across both Farm and Forestry TAPs, regional contexts are consistently related with NBS preparedness, knowledge, and promotion. TAPs working in the Piedmont reported greater confidence and knowledge related to NBS, likely reflecting closer proximity to state agencies, denser professional networks, and stronger technical infrastructure (Knoot and Rickenbach 2014). In contrast, TAPs in the Coastal Plain were positively associated with NBS promotion and implementation, potentially driven by heightened exposure to climate-related risks and adaptation needs such as flooding and erosion (Anderson et al. 2021).

Recent management experience emerged as a consistent predictor across both groups: hands-on activities such as harvesting, replanting, and natural regeneration were positively associated with both preparedness and active promotion, reinforcing evidence that experiential learning plays a central role in building conservation capacity and confidence (Fazey et al. 2006). Together, these findings highlight the importance of regionally tailored capacity-building strategies that leverage recent land-use engagement while addressing professional and institutional barriers to NBS adoption.

### Farm TAPs

Among Farm TAPs, management focus and recent land-use activities were the primary factors associated with perceived preparedness and promotion of NBS. TAPs engaged in forest management and tree planting reported higher preparedness, reflecting alignment with reforestation-oriented NBS objectives, while TAPs managing greater areas of crop and pastureland reported significantly lower preparedness, consistent with the production-oriented nature of these systems and their weaker alignment with conservation practices (Fazey et al. 2006). Recent hands-on experience was strongly correlated with both preparedness and promotion. Positive associations with acres naturally regenerated, replanted, and harvested indicate that direct engagement with land regeneration could enhance confidence and increase the likelihood of active NBS promotion, consistent with findings that experiential learning strengthens conservation capacity (Fazey et al. 2006). In contrast, years employed and age were negatively associated with preparedness and promotion, suggesting that longer-tenured TAPs may be less responsive to emerging conservation approaches or rely more heavily on established management paradigms.

### Forestry TAPs

For Forestry TAPs, sectoral role and professional affiliation were highly correlated with NBS knowledge and preparedness. Government-affiliated TAPs reported significantly higher confidence in promoting NBS, underscoring their role as key intermediaries in translating policy priorities into on-the-ground action through access to technical training, standardized guidance, and institutional mandates emphasizing ecosystem service outcomes (Sagor et al. 2022). Consultants also reported higher preparedness, reflecting their cross-program engagement and applied advisory role (Knoot and Rickenbach 2011). Operational focus further differentiated preparedness. TAPs managing larger forest areas reported greater confidence, suggesting that scale facilitates experiential learning and repeated exposure to NBS practices (Knoot and Rickenbach 2011). Conversely, TAPs focused on general forest management or logging and harvesting revealed a negative association with preparedness, potentially reflecting differences in professional training, incentive structures, or alignment with production-oriented objectives (Joshi and Arano 2009). The negative association between support for expanded carbon markets and perceived preparedness may indicate uncertainty regarding market mechanisms, verification requirements, or long-term viability among practitioners, even when support for NBS is expressed in principle.

### Influence of TAPs’ Perceived Importance of NBS Practices on Active Advocacy and Implementation

The perceived importance of specific forest management objectives was also correlated with TAPs’ promotion of NBS practices. TAPs who placed higher importance on forest establishment, periodic forest management, and site improvement were more closely correlated with actively promoting NBS. This suggests that TAPs who prioritize long-term forest productivity and ecosystem function could be naturally aligned with the goals of NBS, which emphasize sustainability, regeneration, and climate resilience. The positive relationship with carbon offset importance further reinforces the role of climate mitigation as a key motivator for NBS engagement. Interestingly, the perceived importance of general forest maintenance did not correlate with NBS promotion, implying that routine upkeep tasks may not be strongly associated with the broader ecological or climate-focused goals that NBS aims to address. Limited statistical significant results associated with farm practices and the level of importance may be due to the sample size, but further research could provide more insights.

Taken together, our results highlight both distinct and overlapping factors shaping TAPs’ engagement with NBS practices. Across both farm and forestry contexts, regional location consistently influenced priorities, with Piedmont TAPs reporting higher confidence and greater likelihood of promoting NBS, while Coastal Plain TAPs tended to emphasize ecosystem service and general maintenance practices. Practical, regeneration-oriented land management activities were positively associated with both preparedness and active promotion of NBS, suggesting that hands-on experience and observable benefits increase compatibility and reduce uncertainty. Conversely, longer tenure and specialization in production-oriented roles were associated with lower preparedness and promotion, reflecting the greater perceived relative advantage of traditional practices and the institutional inertia often seen among late adopters in DOI theory. In summary, DOI and VBN provide complementary explanations: exposure, observability, and institutional structures influence TAPs’ adoption trajectories, while values and normative commitments determine the strength of their motivation to prioritize and promote NBS.

## Conclusion

This study examines how TAPs’ characteristics, professional affiliations, and organizational sectors shape their perspectives on NBS practices and their broader implementation on working lands. Addressing barriers such as organizational constraints, regional differences, and land-use specific challenges can lead to increased support for broader adoption and effective implementation of NBS. The findings highlight that regional context, professional specialization, and recent management experience were strongly associated with TAPs’ perceptions and confidence, underscoring the need for targeted outreach and capacity-building efforts aligned with local environmental conditions and practitioner expertise. Policies leveraging TAPs’ operational experience, especially in urbanized or environmentally stressed areas, can enhance NBS uptake.

Some limitations of this study should be noted. The relatively small sample size, due to a limited TAP population and partial survey completion, constrained the robustness of quantitative analyses, particularly for farm-focused TAPs. While our results offer valuable insights, there is an opportunity for further research to build on these findings. The sample size also needs to be considered when interpreting farm versus forest results. Although strong support and general acceptance of certain NBS practices exist, barriers remain. Not all practices are equally valued or promoted, likely due to perceived complexity or misalignment with landowners’ priorities. Outreach and educational programs that raise awareness, reduce perceived risks, and enhance TAPs’ understanding of NBS benefits and practical implementation may help overcome these barriers. Expanding TAPs’ capacity in these areas will improve their effectiveness in promoting diverse practices. Investigating the interactions among TAPs, landowners, and policy frameworks will clarify how information and innovations spread, which is critical to scaling climate-smart land management.

Lessons learned in this study such as the importance of TAPs’ operational experience, service regions, and organizational attributes as well as barriers to assisting landowners with adoption of NBS may help inform discussions on NBS policies, incentive programs, and technical assistance. Though accelerated acceptance may still face barriers due to the uncertainty of NBS, similar to what we see internationally, aligning policy tools to address practitioners’ practical challenges can improve NBS adoption, improve ecosystem services, and advance climate mitigation and resilience goals. Equitable funding and training across regions should be considered as it is necessary for equal adoption. Overall, these findings emphasize the importance of policies, outreach, and education tailored to the specific regional, organizational, and operational contexts of TAPs, which are crucial for advancing nature-based solutions. The framework and findings could offer transferable insights for understanding practitioner-driven NBS implementation in other countries and land management systems. As nations work toward climate adaptation and biodiversity commitments under global frameworks such as the Paris Agreement and the Kunming-Montreal Global Biodiversity Framework, insights into how to align policy tools with on-the-ground practitioner realities remain broadly relevant across the globe.

At the same time, these findings should not be interpreted as suggesting that NBS alone can fully address broader structural drivers of environmental degradation or climate vulnerability. In practice, the effectiveness and equity of NBS implementation may depend heavily on long-term institutional support, landowner capacity, funding availability, and broader policy alignment. The variability observed among TAPs in terms of perceived importance, preparedness, and promotion of NBS practices further suggests that adoption is neither uniform nor straightforward. This variability also echoes broader concerns raised by Melanidis and Hagerman (2022), who cautioned that unresolved power dynamics and competing narratives surrounding NBS globally can hinder inclusive participation and limit its transformative potential. These findings reinforce the need to view NBS not as a singular solution, but as one component within a broader portfolio of climate adaptation and land management strategies. Future research should continue examining both the opportunities and limitations of NBS implementation, including how institutional constraints, competing land-use priorities, and socioeconomic factors shape long-term outcomes.

## Data Availability

All data supporting the findings of this study will be available on request to the corresponding author. Data protection and sharing is regulated by an IRB protocol.

## Appendix

Full Results Tables

Tables 6 and 7

**Table 6:** Spearman correlation results (rho) for Farm TAPs practices level of importance

Variable name	Level of importance placed on farm conservation practices	Level of importance placed on farm ecosystem services	Level of importance placed on Managing Carbon	Knowledge and feels equipped to promote NBS practices	Currently promoting NBS practices
	Coefficient (*p* value)	Coefficient (*p* value)	Coefficient (*p* value)	Coefficient (*p* value)	Coefficient (*p* value)
piedmont	−0.4476 (0.0520)	0.2946 (0.2040)	−0.6559 (0.0036)	0.4588 (0.0114)	0.2539 (0.1973)
mountains	0.3038 (0.1864)	0.1333 (0.5738)	−0.0117 (0.9579)	0.3371 (0.0648)	0.0426 (0.8447)
coastalplain	−0.0895 (0.7068)	−0.4714 (0.0377)	0.0416 (0.8622)	0.3162 (0.1055)	0.5000 (0.0095)
FOWFLM	0.1790 (0.4449)	−0.3536 (0.1251)	−0.0746 (0.7564)	0.1147 (0.5774)	0.0907 (0.6535)
FOWFC	−0.4024 (0.0000)	−0.1325 (0.6917)	0.2072 (0.5155)	0.1387 (0.4795)	0.1754 (0.3711)
FOWFRM	−0.3678 (0.1140)	0.3026 (0.1921)	0.0746 (0.7599)	0.4183 (0.0209)	0.5292 (0.0040)
FOWGFM	−0.4476 (0.0470)	0.5303 (0.0182)	−0.0208 (0.9326)	0.6814 (0.0002)	0.5635 (0.0034)
FOWLO	1.0000 (0.0000)	1.0000 (0.0000)	1.0000 (0.0000)	1.0000 (0.0000)	1.0000 (0.0000)
FOWSWC	0.2871 (0.2139)	−0.1260 (0.5932)	0.1548 (0.5242)	0.0000 (0.9722)	0.1195 (0.5576)
FOWTH	−0.1228 (0.5995)	0.4851 (0.0267)	−0.2537 (0.3017)	0.2949 (0.1121)	0.1632 (0.4301)
FOWFHM	−0.1228 (0.5995)	0.0000 (1.0000)	0.0282 (0.9181)	0.2500 (0.1958)	0.3162 (0.0976)
FOWWetM	0.2924 (0.2109)	0.0962 (0.7069)	0.2072 (0.5155)	0.2000 (0.3644)	0.2530 (0.2204)
FOWWLM	−0.2456 (0.3099)	0.4851 (0.0267)	−0.0564 (0.8156)	0.2500 (0.1958)	0.0791 (0.7274)
PFWFM	−0.4385 (0.0655)	0.3849 (0.0915)	−0.3685 (0.1291)	0.2000 (0.3644)	−0.0316 (0.8673)
PFWFPDM	0.2012 (0.6991)	0.2649 (0.3556)	0.2072 (0.5155)	0.1387 (0.4795)	0.1754 (0.3711)
PFWFR	−0.2685 (0.2484)	0.4125 (0.0714)	0.1666 (0.4899)	0.4588 (0.0114)	0.4171 (0.0294)
PFWLH	0.0000 (0.9533)	0.3849 (0.0915)	−0.2010 (0.4306)	0.2500 (0.1958)	0.3162 (0.0976)
PFWP	0.2924 (0.2109)	0.0962 (0.7069)	0.3015 (0.2193)	−0.1000 (0.6108)	−0.0316 (0.8673)
PFWRC	0.2025 (0.3936)	−0.4667 (0.0385)	0.2648 (0.2736)	−0.2697 (0.1879)	−0.1492 (0.4581)
PFWSPTP	−0.4476 (0.0520)	0.0589 (0.8020)	0.1918 (0.4266)	0.4588 (0.0114)	0.4171 (0.0294)
PFWSHEC	0.2025 (0.3936)	−0.4000 (0.0807)	0.2335 (0.3343)	−0.0674 (0.7143)	0.0426 (0.8447)
PFWTM	−0.2644 (0.2549)	0.3482 (0.1311)	−0.0416 (0.8622)	0.5423 (0.0030)	0.3773 (0.0521)
PFWWQ	1.0000 (0.0000)	1.0000 (0.0000)	1.0000 (0.0000)	1.0000 (0.0000)	1.0000 (0.0000)
PFWW	−0.2025 (0.3881)	0.4667 (0.0385)	−0.1284 (0.5959)	0.3371 (0.0648)	0.2345 (0.2393)
SOWFG	−0.1462 (0.5522)	0.0962 (0.7069)	0.3015 (0.2193)	0.2000 (0.3644)	0.2530 (0.2204)
SOWconsultant	−0.0895 (0.6980)	−0.1768 (0.4499)	−0.2395 (0.3181)	0.1147 (0.5774)	0.2539 (0.1973)
SOWNGO	0.2924 (0.2109)	0.0962 (0.7069)	0.0502 (0.8589)	−0.1000 (0.6108)	−0.0316 (0.8673)
SOWPLP	0.0957 (0.6759)	0.1260 (0.5932)	−0.2654 (0.2685)	0.4743 (0.0152)	0.4000 (0.0411)
SOWTIMO	1.0000 (0.0000)	1.0000 (0.0000)	1.0000 (0.0000)	1.0000 (0.0000)	1.0000 (0.0000)
SOWREIT	1.0000 (0.0000)	1.0000 (0.0000)	1.0000 (0.0000)	1.0000 (0.0000)	1.0000 (0.0000)
SOWSG	−0.2924 (0.2471)	0.0962 (0.7069)	0.0502 (0.8589)	0.2000 (0.3644)	−0.0316 (0.8673)
cropandpasturelandmanageda	0.1183 (0.6154)	−0.3945 (0.0854)	−0.2060 (0.3941)	−0.5972 (0.0032)	−0.1899 (0.4192)
percentofmanagedcrop	0.1770 (0.4491)	−0.4583 (0.0434)	−0.1161 (0.6325)	−0.3470 (0.1173)	−0.1792 (0.4354)
percentofmanagedpasture	0.1220 (0.6069)	0.4285 (0.0603)	0.1525 (0.5292)	0.2262 (0.3362)	0.0461 (0.8516)
percentofmanagedwaterandpo	−0.2022 (0.3866)	0.2351 (0.3148)	−0.0262 (0.9122)	0.1623 (0.5170)	0.3867 (0.0742)
percentofmanagedhomesiteb	−0.3843 (0.0974)	0.0990 (0.6743)	−0.2349 (0.3289)	0.3654 (0.0827)	0.1887 (0.4129)
replantedthepastfiveyears	−0.5944 (0.0073)	0.5081 (0.0236)	0.2206 (0.3603)	0.5123 (0.0115)	0.4939 (0.0229)
afforestedinpastfiveyears	−0.3809 (0.1096)	0.1982 (0.4102)	0.4120 (0.0888)	0.3592 (0.0973)	0.3628 (0.1080)
regeneratenaturallypastfivey	−0.4430 (0.0541)	0.5084 (0.0237)	0.0342 (0.8892)	0.3642 (0.0831)	0.1035 (0.6634)
havestedinpastfiveyears	−0.5909 (0.0075)	0.4395 (0.0534)	0.0920 (0.7052)	0.5438 (0.0067)	0.4739 (0.0304)
yearsemployedmid	0.3276 (0.1586)	−0.4178 (0.0675)	−0.1847 (0.4447)	−0.3922 (0.0690)	−0.4075 (0.0695)
orgsize_mid	−0.2405 (0.3171)	0.3596 (0.1296)	0.2600 (0.2943)	0.3003 (0.2006)	0.3256 (0.1592)
Age	0.4205 (0.0660)	−0.2742 (0.2390)	−0.0102 (0.9667)	−0.3890 (0.0878)	−0.2347 (0.3157)
Education	−0.1570 (0.5054)	−0.1469 (0.5339)	−0.0850 (0.7275)	0.0396 (0.8688)	0.0925 (0.6955)

**Table 7:** Spearman correlation results (Rho) for forest TAPs practices level of importance

Variable	Level of importance forest establishment	Level of importance periodic forest management *Coefficient*	Level of importance general forest maintenance *Coefficient*	Level of importance site improvement *Coefficient*	Level of importance carbon offsets *Coefficient*	NBSknowledge rho (p)	NBSpromoting rho (p)
	*Coefficient*	*(p value)*	*(p value)*	*(p value)*	*(p value)*		
	*(p value)*						
piedmont	−0.0978 (0.3958)	−0.0220 (0.8473)	−0.1814 (0.1094)	−0.2808 (0.0127)	−0.2538 (0.0253)	0.2940 (0.0034)	0.1949 (0.0588)
mountains	0.0391 (0.7376)	−0.0129 (0.8854)	−0.1796 (0.1141)	0.0503 (0.6610)	0.1370 (0.2312)	0.1589 (0.1151)	0.0306 (0.7663)
coastalplain	0.0272 (0.8140)	0.2309 (0.0383)	0.3077 (0.0062)	−0.0183 (0.8721)	−0.0304 (0.7909)	0.0720 (0.4900)	0.0654 (0.5268)
FOWFLM	0.1946 (0.0833)	0.1135 (0.3190)	−0.0388 (0.7345)	0.0963 (0.4119)	0.0790 (0.5004)	0.1332 (0.1691)	0.1540 (0.1434)
FOWFC	0.0812 (0.4952)	0.1252 (0.2497)	0.1888 (0.0915)	0.1826 (0.1029)	0.0608 (0.6047)	0.0653 (0.5753)	0.1359 (0.1958)
FOWFRM	0.0475 (0.6839)	0.1873 (0.0795)	0.0227 (0.8443)	0.0400 (0.7284)	0.1746 (0.1263)	0.0900 (0.3905)	0.2020 (0.0504)
FOWGFM	−0.1679 (0.1424)	0.0279 (0.7714)	−0.0985 (0.3883)	−0.1327 (0.2432)	-0.0051 (0.9646)	0.1736 (0.0991)	0.1971 (0.0551)
FOWLO	0.0124 (0.9245)	–0.3100 (0.0159)	−0.2906 (0.0094)	−0.0444 (0.6953)	−0.0265 (0.8206)	−0.0056 (0.9042)	-0.0599 (0.5741)
FOWSWC	0.1920 (0.0897)	0.1361 (0.1974)	−0.0129 (0.9053)	0.0268 (0.8255)	0.0667 (0.5663)	0.1594 (0.0873)	0.2166 (0.0343)
FOWTH	−0.1176 (0.3069)	−0.1212 (0.2861)	−0.0519 (0.6484)	−0.0024 (0.9825)	−0.1229 (0.2826)	0.0956 (0.3569)	−0.0605 (0.5588)
FOWFHM	−0.0608 (0.5969)	0.0495 (0.6865)	0.0054 (0.9643)	0.0863 (0.4496)	0.2237 (0.0492)	0.1024 (0.3244)	0.0792 (0.4446)
FOWWetM	0.1076 (0.3758)	0.0868 (0.5586)	−0.1194 (0.3150)	−0.0829 (0.4864)	0.0310 (0.7971)	0.1021 (0.3528)	0.1903 (0.0616)
FOWWLM	0.0413 (0.7295)	−0.1329 (0.2571)	-0.1831 (0.1090)	0.1255 (0.2721)	−0.0508 (0.6614)	0.1138 (0.2706)	−0.0506 (0.6333)
PFWFM	0.0474 (0.6869)	0.1157 (0.3092)	0.1060 (0.3536)	−0.0837 (0.4624)	−0.1118 (0.3306)	0.1230 (0.2294)	0.0237 (0.8177)
PFWFPDM	−0.0923 (0.4262)	0.0511 (0.7163)	−0.0594 (0.6028)	−0.0010 (0.9865)	0.1342 (0.2436)	0.1939 (0.0336)	0.1555 (0.1354)
PFWFR	0.1203 (0.2960)	0.0461 (0.7012)	−0.0465 (0.6822)	0.1955 (0.0836)	0.3932 (0.0004)	0.1725 (0.0881)	0.2172 (0.0353)
PFWLH	−0.1997 (0.0821)	−0.0889 (0.4324)	−0.0332 (0.7700)	−0.1083 (0.3407)	−0.2490 (0.0283)	−0.0401 (0.6938)	−0.0430 (0.6779)
PFWP	0.1198 (0.4447)	0.0495 (0.3726)	−0.0681 (0.6780)	−0.0708 (0.6688)	0.1059 (0.4787)	0.0583 (0.5719)	0.1087 (1.0000)
PFWRC	0.1706 (0.1315)	0.0704 (0.8137)	0.0368 (0.7903)	0.1667 (0.1395)	−0.0377 (0.7685)	0.0829 (0.5626)	0.1546 (0.1557)
PFWSPTP	−0.0226 (0.8430)	0.0794 (0.4910)	0.1649 (0.1455)	−0.0769 (0.4984)	−0.0587 (0.6083)	0.1144 (0.2683)	−0.0046 (0.9652)
PFWSHEC	0.3036 (0.0036)	0.1252 (0.2497)	0.1448 (0.2053)	0.0586 (0.6214)	0.1174 (0.3122)	0.1468 (0.1210)	0.1870 (0.0709)
PFWTM	−0.1310 (0.2552)	−0.0761 (0.5218)	−0.0413 (0.7198)	−0.0599 (0.6021)	−0.0472 (0.6810)	0.0918 (0.3744)	0.0095 (0.9277)
PFWWQ	0.1944 (0.0864)	0.1565 (0.1255)	−0.1016 (0.3760)	0.0901 (0.4355)	−0.0438 (0.7062)	0.0989 (0.3504)	0.0530 (0.6125)
PFWW	−0.1061 (0.3593)	−0.0142 (0.8454)	−0.2120 (0.0624)	0.0136 (0.9112)	0.0165 (0.8858)	0.0670 (0.5447)	−0.0434 (0.6813)
SOWFG	0.0812 (0.4952)	0.1361 (0.1974)	0.0149 (0.9058)	0.0586 (0.6214)	0.1352 (0.2427)	0.1594 (0.0873)	0.2973 (0.0023)
SOWconsultant	−0.0634 (0.5815)	−0.0469 (0.6653)	−0.2222 (0.0500)	−0.1024 (0.3677)	−0.0109 (0.9249)	0.1498 (0.1411)	0.1728 (0.0946)
SOWNGO	0.0049 (0.9863)	−0.1135 (0.3394)	−0.1895 (0.1010)	0.0956 (0.4300)	−0.0465 (0.7028)	0.1185 (0.2404)	0.0110 (0.9131)
SOWPLP	−0.2512 (0.0277)	−0.0561 (0.6270)	−0.0478 (0.6748)	−0.2354 (0.0369)	0.0306 (0.7893)	0.1169 (0.2587)	0.0732 (0.4786)
SOWTIMO	−0.0483 (0.6773)	0.1135 (0.3190)	0.2750 (0.0086)	0.0963 (0.4119)	−0.1143 (0.3273)	−0.0287 (0.7374)	−0.1062 (0.3142)
SOWREIT	0.0282 (0.8214)	0.1252 (0.2497)	0.0149 (0.9058)	−0.1196 (0.3015)	−0.1654 (0.1496)	−0.0287 (0.7374)	−0.0255 (0.8161)
SOWSG	0.1431 (0.2134)	0.1478 (0.1772)	−0.0222 (0.8429)	0.1201 (0.2917)	0.0527 (0.6467)	0.1356 (0.1809)	0.0521 (0.6152)

## Footnotes

1 Full results of Fig. 2 are presented in Table 6 in the appendix

2 A full table is available in the appendix (Table 7)

3 This is not reported in the table due to only having one significant variable, it is included in the Appendix. (Table 7)
